# Prediction model with multi-point relationship fusion via graph convolutional network: A case study on mining-induced surface subsidence

**DOI:** 10.1371/journal.pone.0289846

**Published:** 2023-08-16

**Authors:** Baoxing Jiang, Kun Zhang, Xiaopeng Liu, Yuxi Lu

**Affiliations:** 1 State Key Laboratory of Mining Response and Disaster Prevention and Control in Deep Coal Mines, Huainan, China; 2 School of Geomatics, Anhui University of Science and Technology, Huainan, China; 3 School of Information Science and Engineering, Shandong Normal University, Jinan, China; 4 Faculty of Land Resources Engineering, Kunming University of Science and Technology, Kunming, China; TU Wien: Technische Universitat Wien, AUSTRIA

## Abstract

Accurate prediction of surface subsidence is of significance for analyzing the pattern of mining-induced surface subsidence, and for mining under buildings, railways, and water bodies. To address the problem that the existing prediction models ignore the correlation between subsidence points, resulting in large prediction errors, a Multi-point Relationship Fusion prediction model based on Graph Convolutional Networks (MRF-GCN) for mining-induced subsidence was proposed. Taking the surface subsidence in 82/83 mining area of Yuandian No. 2 Mine in Anhui Province in eastern China as an example, the surface deformation data obtained from 250 InSAR images captured by Sentinel-1A satellite from 2018 to 2022, combined with GNSS observation data, were used for modeling. The deformation pattern of each single observation point was obtained by feeding their deformation observation data into the LSTM encoder, after that, the relationship graph was created based on the correlation between points in the observation network and MRF-GCN was established. Then the prediction results came out through a nonlinear activation function of neural network. The research shows that the *R*^2^R2 value of MRF-GCN model was 0.865 0, much larger than that of Long-Short Term Memory (LSTM) and other conventional models, while mean square error (MSE) of MRF-GCN model was 1.59 899, much smaller than that of LSTM and other conventional models. Therefore, the MRF-GCN model has better prediction accuracy than other models and can be applied to predicting surface subsidence in large areas.

## 1 Introduction

Surface subsidence is a common geological environmental disaster, which is increasingly concerned [1–4]. It is characterized by a long development cycle, irreversibility, and continuous accumulation of destructive effects, that usually brings a series of unfavorable impact on people’s production and daily life [5–9]. Especially surface subsidence caused by underground coal mining may lead to irreparable damage to buildings, roads and farmland, etc. in mining areas [10, 11]. Prediction with high accuracy contributes to the prevention and control of the damage caused by disaster such as surface subsidence in large-scale regions.

The existing models for predicting surface subsidence are mainly divided into two types: physical models and statistical models [12–14]. Physical models [15–18] are established according to the mechanical properties of different rock layers to predict the surface subsidence, of which the limitation is that the strong dependence on the characteristics of geological structure in the study area what makes it difficult for them to form a unified and universal framework. Statistical models [14, 19] obtain regular patterns through the analysis of a large amount of existing data, and based on this, predict the future trend of the data. The statistical model requires the acquisition of strict sedimentation data, which has very high requirements on the acquisition of data, especially in the past, when manual observation was time-consuming, laborious, and inefficient, making it difficult to obtain data and predict surface subsidence in large areas. With the development of remote sensing technology [20–22] and deep learning [23], acquisition and processing of long-term, large-scale surface deformation data was no longer a problem. But there is an increasing demand for data mining accuracy.

The emergence of Graph Convolutional Network (GCN) [24–26] is well suited to meet the requirements of accurate data mining. GCN is an practical variant of the traditional convolutional neural network (CNN) [27] proposed by Kipf and Welling [24]. GCN can effectively perform convolution operations on irregular topological graph structure information, thereby learning the hidden information associated with the graph interior. Taking surface subsidence as an example, each observation point does not exist independently, and there are strong internal connections between them. Based on these connections, the pattern of surface subsidence can be obtained.

In general, the graph structure data consists of a set N of nodes and a set E of edges, where $N\in R^{n\times m}$ stores the *n* nodes of the graph structure, and the information of each node is uniquely represented by an *m*-dimensional vector, and the set of edges $E\in R^{n\times n}$ stores the connection relations between all nodes in the graph structure, represented by an *n* dimensional symmetric 0 − 1 matrix *A*, which is usually referred to as the adjacency matrix. The adjacency matrix *A* is generated by the following equation.

$$\begin{array}{c} \left\{ \begin{array}{c} A_{ij}=1,\;if\;the\;i_{th}\;node\;is\;connected\;to\;the\;j_{th}\;node;\\ A_{ij}=0,\;otherwise; \end{array}\right. \end{array}$$ (1)

GCN can aggregate local information between neighboring nodes by performing convolution operations on the graph. When performing multi-layer convolution operations, the information in the graph can be passed farther through the connections between nodes, achieving the purpose of fusing local and global information. The following equation updates the hidden state $h_{i}^{l}$ of the *i* − *th* node in the *l* − *th* layer.

$$\begin{array}{c} h_{i}^{l}=\sigma (\sum_{j=0}^{n}A_{ij}W^{l}h_{j}^{l-1}+b^{l}) \end{array}$$ (2)

In the Eq 2, *W*^*l*^ is the weight matrix, $h_{j}^{l-1}$ is the hidden state of the *j* − *th* node in the *l* − 1th layer, *b*^*l*^ is the bias, and *σ* is the nonlinear activation function (e.g., ReLU). When *l* = 1, the hidden feature $h_{j}^{0}$ of the node takes the initial state of the node.

Taking the surface subsidence in 82/83 mining area of Yuandian No. 2 Mine in Anhui Province in eastern China as an example, the surface deformation data obtained from 250 InSAR images captured by Sentinel-1A satellite from 2018 to 2022, combined with GNSS observation data, were used for establishing a prediction model with MRF-GCN. The main contributions of this paper are as follows.

1. We design and implement a prediction model MRF-GCN for surface subsidence based on graph convolutional networks. The model runs a graphical convolution operation in the surface subsidence monitoring network, which can model the interaction between monitoring points due to geological heterogeneity and thus make more accurate predictions of subsidence at surface points.
2. We successfully predicted the surface subsidence pattern in a mining area in China by the model proposed in this paper and elaborated on the principles of the model work. Meanwhile, we designed ablation experiments to confirm the effectiveness of the model modules.
3. We write the code based on the Python language and implement it based on the latest Pytorch deep learning architecture. To facilitate related research, our code is publicly available at https://github.com/BaoSir529/MRF-GCN

## 2 Methodology

**Fig 1:**
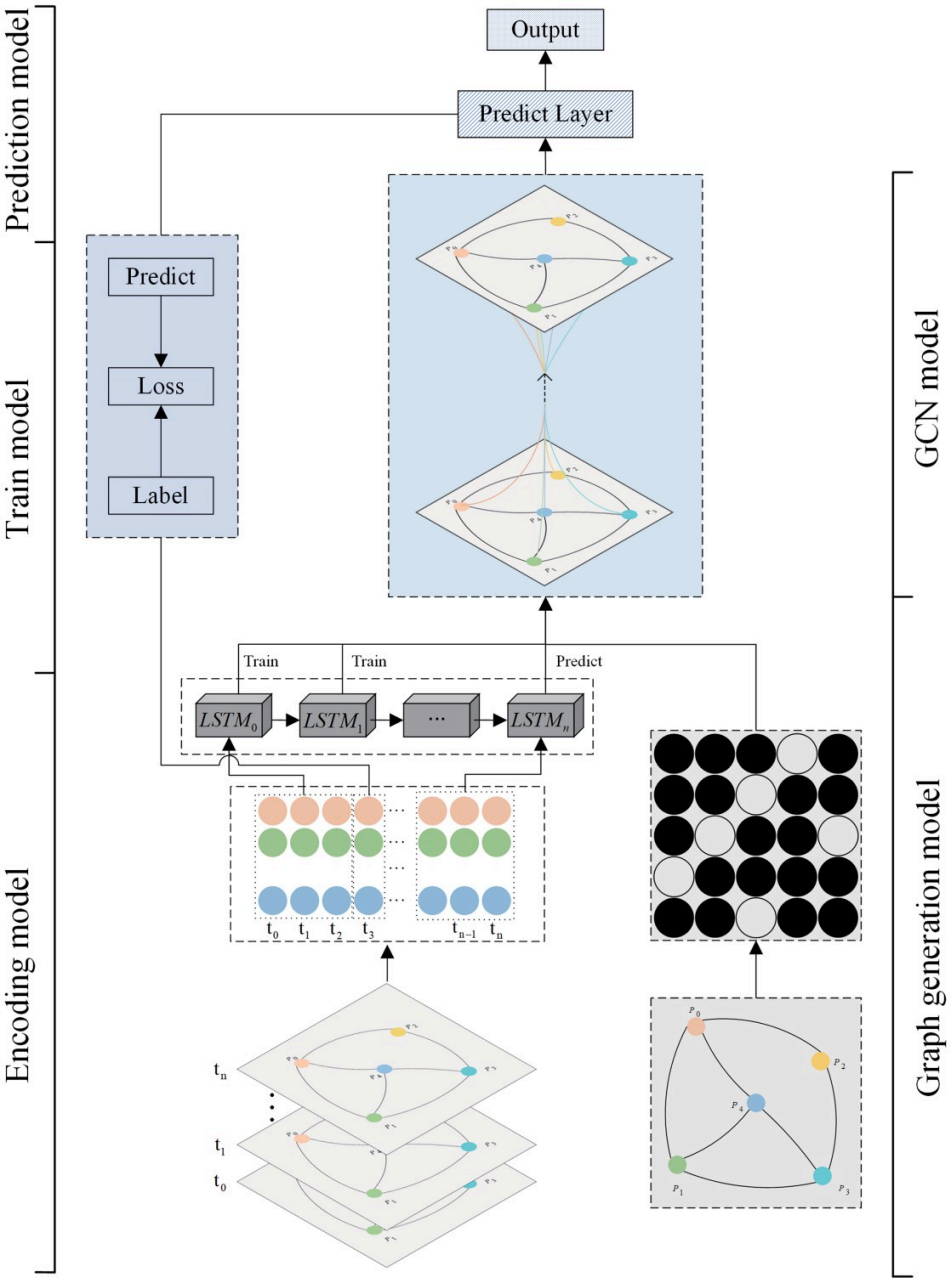
Model architecture. MRF-GCN consists of five parts: Graph generation module, Encoding module, GCN module, Training module, and Prediction module.

As shown in Fig 1, MRF-GCN consists of five parts: graph generation module, encoding module, graph convolutional network (GCN) module, training module, and prediction module. Among them, the encoding module is designed to learn the contextual information of the time series data based on the LSTM model, and the graph generation module generates the graph information for the graph convolution operation based on the association between the target points in a given region, the graph convolution module is designed to receive the encoding information and the graph information for the convolution operation, the training module is responsible for generating a pre-trainedpre-training model, and the prediction module will output the final prediction based on the pre-trained model.

### 2.1 Task defination

In the case of large-scale surface subsidence monitoring, denote a series of surface monitoring points as *P* = {*p*_0_, *p*_1_, …, *p*_*n*_}. For a given surface monitoring point, there are continuous time series data *t*_*i*_ = {*t*_0_, *t*_1_, …, *t*_*n*_}. Depending on the type of monitoring, these time-series data can provide feedback on changes in data at the site over time (e.g., elevation, soil temperature, humidity). The surface subsidence prediction task aims to predict data for a future period based on a priori data *t*_*i*_, i.e., to predict {*t*_*n*+1_, *t*_*n*+2_, …., *t*_*n*+*k*_}.

### 2.2 Graph generation module

In order to obtain subsidence data for a particular monitoring area, a monitoring network needs to be set up several years in advance, and the observation area needs to be continuously observed for an extended period by manual level measurement or in combination with remote sensing technology. In this paper, we selected the surface of the 82/83 mining area of Yuandian No. 2 Mine in Anhui Province, China, as the observation area. We set up the monitoring network in advance and obtained the continuous deformation data in this area by manual level measurement and InSAR image-based extraction. In order to confirm the reliability of the model, we select two observation networks with 20 monitoring points in the manual measurement area and the InSAR image processing area, respectively, as the demonstration objects of this paper.

According to China National General Specification for Engineering Surveying requirements, we select the monitoring points within the artificial measurement area. In addition, when selecting points, avoid extremely sloping ground and water and choose sites that can be preserved for a long time and are easy to observe. The relationship between monitoring points in all observation networks follows the following guidelines.

1. More excellent connectivity between monitoring sites in close spatial proximity.
2. More excellent connectivity of monitoring points on the same geological structure.
3. Lack of connectivity between monitoring sites isolated by large geological structures (e.g., hills, lakes) or building complexes.
4. The connection between monitoring points should consider the actual placement of the level observation network.

**Fig 2:**
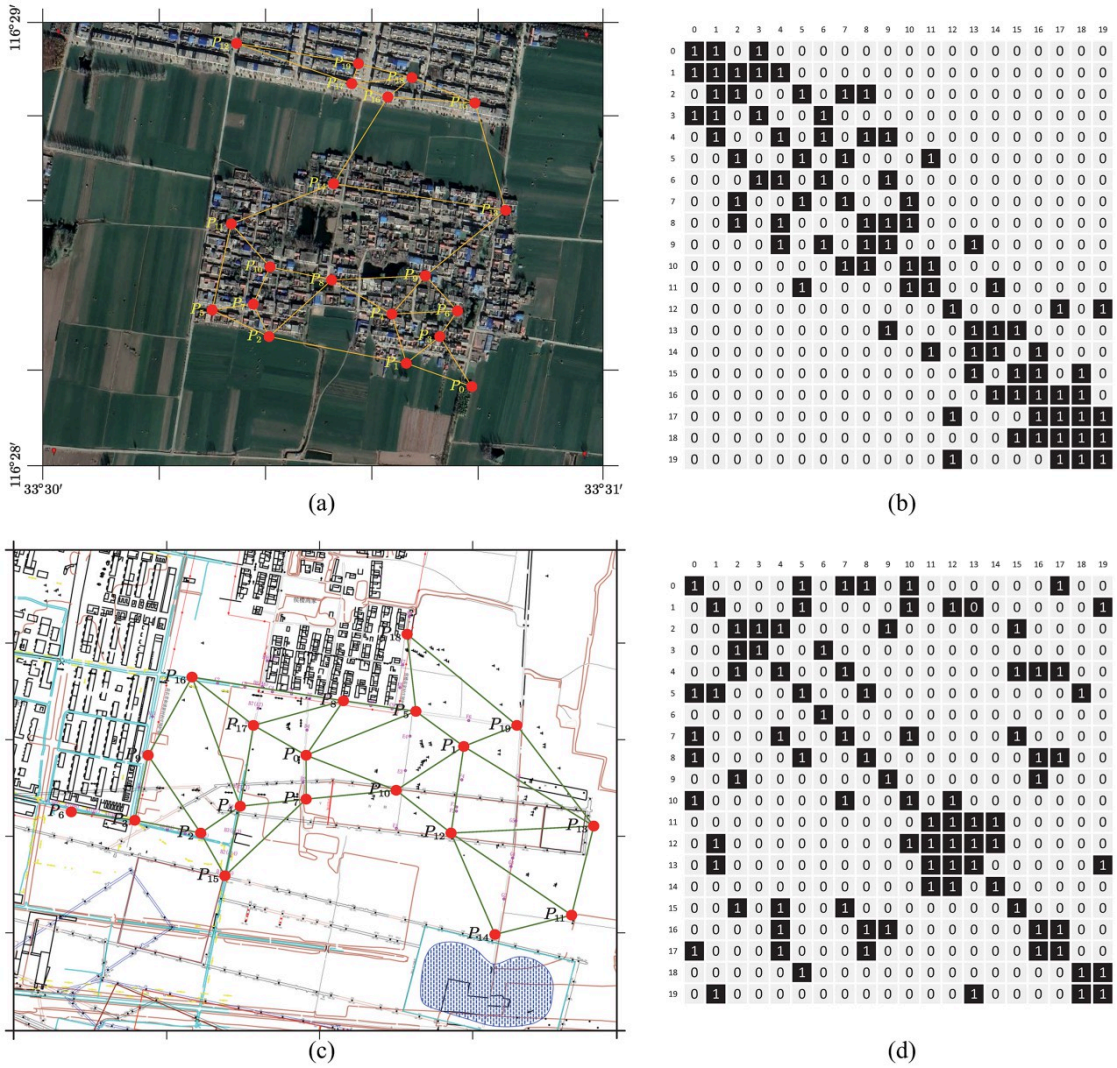
Study area and point layout map. (a) is the InSAR-based control network, (b) is the corresponding adjacency matrix; (c) is the manual-based control network, and (d) is the corresponding adjacency matrix. InSAR images reprinted from Earth Resources Observatory and Science (EROS) Center under a CC BY license. InSAR image obtained from the Earth Resources Observatory and Science (EROS) Center (http://eros.usgs.gov).

The control network is laid in the remote sensing area, and the manual measurement area is shown in Fig 2. We obtain the adjacency matrix of the corresponding regional monitoring network according to the Eq 1 and the above criterion. For convenience, the adjacency matrices based on manual leveling and InSAR images are unified as *A*, and the subsequent contents do not distinguish between the two regions.

### 2.3 Encoding module

After continuously observing monitoring points *P* = {*p*_0_, *p*_1_,., *p*_*n*_}, each monitoring point *p*_*i*_ obtains a time series information reflecting its elevation change $t^{i}=\left\{t_{0}^{i},t_{1}^{i},\ldots ,t_{n}^{i}\right\}$. In order to train the model to learn the settlement pattern at each monitoring point, a Long Short-Term Memory (LSTM) [28, 29] network module is used as an encoder to train the model to learn the settlement pattern at different monitoring points before and after time nodes. LSTM is an implementation of the Gated Recurrent Neural network (GRU) [30], which can effectively obtain the changing pattern before and after the sequence data.

To achieve the goal of learning future subsidence patterns using a priori sequences, we set up a window (window) that allows us to look forward at *k* steps of the preliminary data and train the model to learn the patterns of all sequential data by scanning the prior data item by item with the sliding of this window. The specific experimental procedure of this process is that, first, the sequence data of each monitoring point in the control network is divided into overlapping data segments according to the window width *K*. For a monitoring point *p*_*i*_ the time series information is divided into $\left\{\left(t_{0}^{i},\ldots ,t_{k}^{i}\right),\left(t_{1}^{i},\ldots ,t_{k+1}^{i}\right),\ldots ,\left(t_{n-k}^{i},\ldots ,t_{n}^{i}\right)\right\}$, we can get *n* − *k* + 1 data segments, notated as $x^{i}=\left\{x_{0}^{i},x_{1}^{i}\ldots .x_{n-k}^{i}\right\}$. For all monitoring points in the detection network, the input data generated at each step after the window scan is $X\in R^{n\times k}$. Second, we feed the data segments generated from all monitoring points in the detection network one by one into a *l*-layer LSTM module, with each segment of length *k* as an input state at the current time *t*. For each monitoring point input, the following function is computed for each layer of the LSTM module.

$$\begin{array}{c} I_{t}=\sigma (W_{0}x_{t}+b_{0}+W_{1}h_{t-1}+b_{1}) \end{array}$$ (3)

$$\begin{array}{c} F_{t}=\sigma (W_{2}x_{t}+b_{2}+W_{3}h_{t-1}+b_{3}) \end{array}$$ (4)

$$\begin{array}{c} G_{t}=\text{tanh}(W_{4}x_{t}+b_{4}+W_{5}h_{t-1}+b_{5}) \end{array}$$ (5)

$$\begin{array}{c} O_{t}=\sigma (W_{6}x_{t}+b_{6}+W_{7}h_{t-1}+b_{7}) \end{array}$$ (6)

$$\begin{array}{c} C_{t}=F_{t}⊙C_{t-1}+I_{t}⊙G_{t} \end{array}$$ (7)

$$\begin{array}{c} h_{t}=O_{t}⊙\text{tanh}(C_{t}) \end{array}$$ (8)

where *h*_*t*_ is the hidden state at time *t*, *h*_*t*−1_ is the hidden state of the layer at time *t* − 1 or the initial hidden state at time 0, *C*_*t*_ is the cell state at time *t*, *x*_*t*_ is the input at time *t*, and *I*_*t*_, *F*_*t*_, *G*_*t*_, *O*_*t*_ are the input, forget, cell, and output gates, respectively. ⊙ is the Hadamard product. *W*_*i*_ and *b*_*i*_ is the corresponding parameter matrix and bias. In a multilayer LSTM, the input $x_{t}^{\left(l\right)}$ of the *l* − *th* layer is the hidden state $h_{t}^{\left(l-1\right)}$ of the previous layer. After *l* layers of the LSTM module, for all monitoring points in the detection network, the hidden state $h_{t}^{\left(l\right)}$ of each time point in the last layer is taken to get the LSTM encoded state *H*^*lstm*^.

Finally, to facilitate the subsequent graph convolution operation, the subsidence features extracted by LSTM are subjected to nonlinear changes to extract the association information between them and finally mapped to a new feature space. Their spatial dimension is set as the input dimension of the graph convolution module.

$$\begin{array}{c} H=WH^{lstm}+b \end{array}$$ (9)

The final coded information $H\in R^{n\times c}$ is obtained for each monitoring point in *k* consecutive a priori monitoring data, where *c* is the input dimension of the graph convolution module, W and b are the parameter matrix and bias of the nonlinear transformation.

### 2.4 GCN module

The observation area of the continuous surface subsidence monitoring task is large, and the geographical factors are complex. The points in the monitoring network interact with each other and have complex intrinsic interactions. For example, changing points in the monitoring network within the area to be trapped can continuously affect surrounding points. In order to better tap the interaction pattern between points, we introduce a designed graph convolution module. By modeling based on the graph structure information of the monitoring network and the information of the continuous settlement pattern of the monitoring points, it helps the model to make a more optimistic prediction of the surface settlement.

After the LSTM coding module, the model obtains the coding information of each monitoring point in the monitoring network about the continuous *k* a priori data. In the actual monitoring, the monitoring data of each monitoring point in the monitoring network are obtained in the same period and independently measured. This pattern is often reflected in the monitoring data of the duration when the monitoring points in the same monitoring network interact with each other. According to the graph generation module and LSTM coding module mentioned above, we obtain the adjacency matrix *A* of points within the monitoring network and the continuous variation law *H* of each monitoring point fed into a designed graph convolutional neural network.

To eliminate the effect of non-normalization of the monitoring point relationship map, we normalize the point adjacency matrix according to the suggestion of Kipf & Welling [24].

$$\begin{array}{cc} & e=D^{-\frac{1}{2}}\hat{A}D^{-\frac{1}{2}} \end{array}$$ (10)

$$\begin{array}{cc} & D_{ii}=\sum_{j}A_{ij} \end{array}$$ (11)

$\hat{A}=A+I$ denotes the self-loop of each node, *I* is a unit matrix, and *D* is the degree matrix of *A*, storing the number of nodes that each node joins to the rest.

Next, the GCN updates the state information of each monitoring point in layer *l* by aggregating the state information of neighboring monitoring points in layer *l* − 1, and the network is operated according to the following function.

$$\begin{array}{c} s_{p_{i}}^{\left(l\right)}=ReLU\left(\sum_{j}\frac{1}{e_{ij}}s_{p_{j}}^{\left(l-1\right)}W^{l-1}\right) \end{array}$$ (12)

where $s_{p_{i}}^{\left(l\right)}$ denotes the output of node *p*_*i*_ at the *l*-th layer of GCN. $s_{p_{j}}^{\left(l-1\right)}$ represents the node *p*_*j*_’s neighbor node *p*_*i*_’s output at the *l* − 1-th layer of GCN, and also as the input of the *l*-th layer. It should be noted that when *l* = 1, the input state of the GCN is the initial state of the neighbor node *h*_*j*_, where *h*_*j*_ ∈ *H*. *e*_*ij*_ is a normalization constant for the edge (*p*_*i*_, *p*_*j*_) which originates from using the symmetrically normalized adjacency matrix $D^{-\frac{1}{2}}\hat{A}D^{-\frac{1}{2}}$. *W* is a nonlinear parameter matrix.

### 2.5 Training and optimization

The training of a neural network is continuously adjusting the weight matrix while minimizing the error function by back-propagating the gradient. In order to fully utilize the limited data in the dataset, the error profile of the model can be trained to converge by setting multiple epochs. In order to obtain the predictions of the model from the priori data of each monitoring point, we designed a Feed forward neural network (FFNN) immediately following the GCN module as the prediction output layer, and the predicted values are given according to the following functions.

$$\begin{array}{c} \hat{y}=FFNN\left(s_{p_{i}}\right) \end{array}$$ (13)

where $s_{p_{i}}$ is the output of the monitoring point *p*_*i*_ after the graph convolution module, and $\hat{y}$ is the predicted value at time *k* + 1 made by the model based on the monitoring point a priori data.

In terms of the error function, the mean square error of the predicted value and the target value is chosen as the error function in this paper.

$$\begin{array}{cc} Loss & =MSE(y,\hat{y}) \end{array}$$ (14)

$$\begin{array}{c} =\frac{1}{n}\sum_{t=1}^{n}{\left(y_{t}-{\hat{y}}_{t}\right)}^{2} \end{array}$$ (15)

where $\hat{y}$ and *y* are the predicted and true values of the monitoring points, respectively. Adam is chosen as the optimizer of the model, and the goal of model training is to make the error function tend to be minimized.

## 3 Experimental results and analysis

### 3.1 Study area and data sources

**Fig 3:**
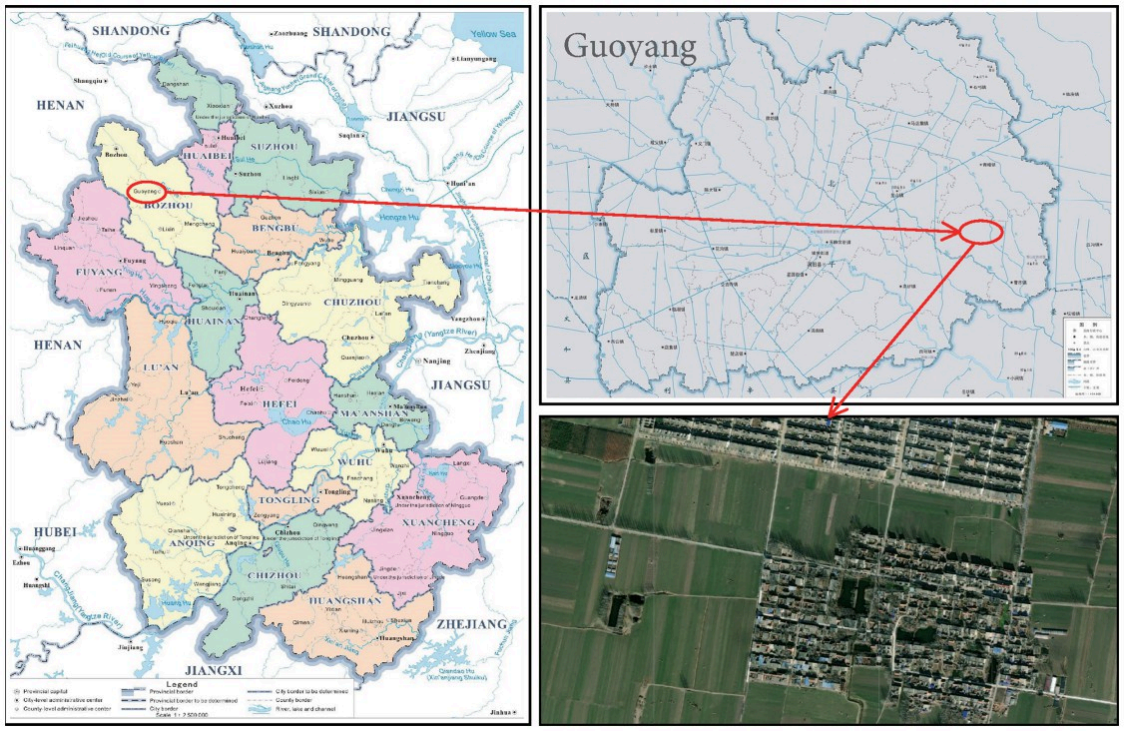
Schematic diagram of the study area. InSAR image reprinted from Earth Resources Observatory and Science (EROS) Center under a CC BY license. Map source: Anhui Provincial Department of Natural Resources, Bozhou Natural Resources and Planning Bureau. Insar image of the study area obtained from the Earth Resources Observatory and Science (EROS) Center (http://eros.usgs.gov).

The study area of this paper is mainly the 82/83 mining area of Yuandian No. 2 Mine in Anhui Province and the surrounding collapse area in Anhui Province, China, which is located in the northern part of Anhui Province, and its center is about 55*km* from Suizhou City in the east and 52*km* from Huaibei City in the northeast. Geographical coordinates are 116°23′59′′*E* ∼ 116°32′04′′*E*, 33°29′05′′*N* ∼ 33°33′55′′*N*. The east-west length is about 10.9*km* ∼ 13.3*km*, the north-south width is about 1.3*km* ∼ 5.3*km*, the area is about 41.6*km*^2^, and the geographical location is shown in Fig 3. The mine was built in 2007 and started production in 2011. The mining depth is −250*m* ∼ −1000*m* elevation, the main mining 7_2_ coal seam, coal thickness 0*m* ∼ 5.7*m*, coal mining mainly according to the directional longwall full trap collapse method.

To obtain remote sensing image-based subsidence data for the study area, we used remote sensing images taken by the C-band Sentinel-1A satellite of the European Space Agency. From January 2018 to December 2022, 148 SAR images were acquired with an average interval of 12 days, a polarization of VV+VH, and a satellite attitude of ascending orbit phase at the time of photography. Based on the original image data, we use multiple Master-image Coherent Target Small-baseline Interferometric SAR method [31, 32] to generate InSAR deformation time series data. For the missing values due to coherence, the local linear interpolation method is used to fill in the missing values. The time baseline was set to 48 days, and the spatial baseline was 150 m. A total of 391 baseline pairs were formed. Finally, a total of 127,000 time series sedimentation values of points were obtained, from which 20 target points were selected as the data source of this paper, and 146 sedimentation data of the same time interval were obtained for each target point. In addition, to eliminate the influence of systematic errors and topographic phases on the deformation results, we also obtained the corresponding accurate orbital ephemeris and elevation data to correct the orbital information. It should be noted that there are more than one method for obtaining deformation data based on InSAR images, and it needs to choose the method according to actual situations to better reflect the trend of surface subsidence.

Further, in order to obtain more accurate field data, we set up a long-term surface settlement monitoring network with 64 monitoring points in conjunction with the current level points in the mine site. From September 2017 to October 2018, with an average interval of 10 days, the monitoring points were measured at the third-order leveling following China’s “Specifications for the third and fourth order leveling”. Similarly, 20 monitoring points uniformly covering the mining plane of the mine area were selected from the measurement results as a manual data source with 37 consecutive data for each subsidence sequence.

### 3.2 Model hyperparameter setting

**Fig 4:**
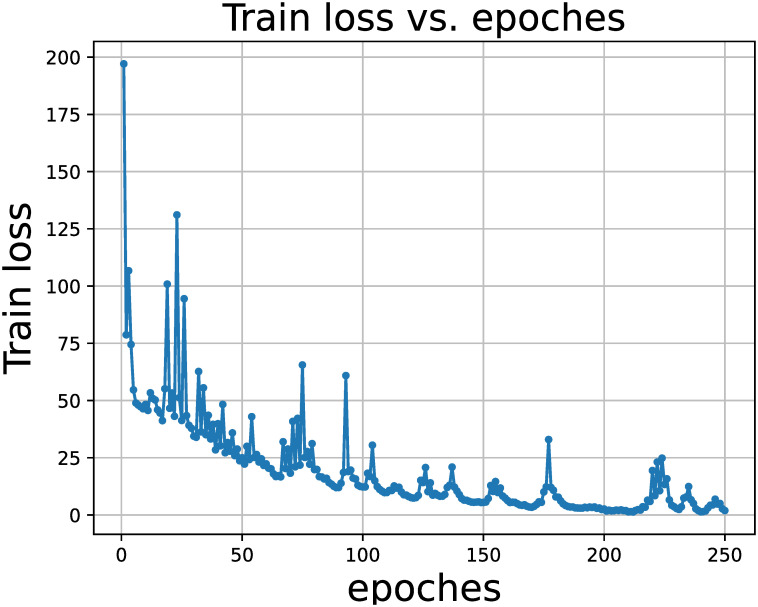
Training loss variation. The model converges in the region of 180 to 220 epochs.

Our model is trained on an NVIDIA GeForce RTX 3090Ti with sliding window set to 5, and LSTM module hidden state dimension is 300, and GCN module hidden state set to 300. The experimental data set was split according to 80% as the training set and the remaining 20% as the test set. Too little training does not allow the model to converge, while too much training introduces additional noise. As shown in Fig 4, the model converges in the region of 180 to 220 epochs, so we choose to set the training epoch to 200. According to the actual training platform, we set the batch size of per epoch to 20.

### 3.3 Results and analysis

To verify the accuracy of the model prediction, we use the first 80% data of the time series data as the training set, train the model to predict the subsequent data, and compare it with the real data. Meanwhile, to verify the advancedness of MRF-GCN, we conducted controlled tests using three common time-series prediction models, all benchmark tests used the same data sources, and all models simultaneously predicted future settlement changes at all monitoring sites in the monitoring network.

- Autoregressive integrated moving average (ARIMA) [33] is one of the most commonly used time series forecasting models that uses first-order differences to smooth the original data and selects the model parameters (*p*, *q*) as (1, 2) according to the data specifics.
- Space-time ARIMA (STARIMA) [34] is a variant of the ARIMA model, which performs multi-point forecasting by constructing an ARIMA model for each point location.
- LSTM [28] is a practical variant of recurrent neural networks. It is a typical deep learning method for surface sedimentation prediction tasks using LSTM as an encoder to learn the temporal patterns of sedimentation sequences.

Finally, the statistical results between all model predictions and the actual values are presented based on the monitoring network data obtained from InSAR images. The Pearson correlation coefficient (PCC) can describe the degree of linear correlation between two matrices, close to 1. It indicates that the two matrices have a strong linear correlation. The *R*^2^ characterizes the degree of correlation between the two data sets, and the mean square error (MSE) can reflect the degree of dispersion between the two data sets. The smaller it is, the better the two data sets match. The experimental results of each model are shown in Table 1.

**Table 1:** Comparison of experimental results of different prediction models. PCC is the Pearson correlation coefficient, and MSE means mean square error.

Model	PCC	*R* ^2^	MSE/*mm*^2^
ARIMA(1,1,2)	0.2413	-4.9451	11.5866
STARIMA	0.7114	0.1325	14.7639
LSTM	0.8899	0.4572	3.0151
MRF-GCN	0.9329	0.8650	1.5989

**Fig 5:**
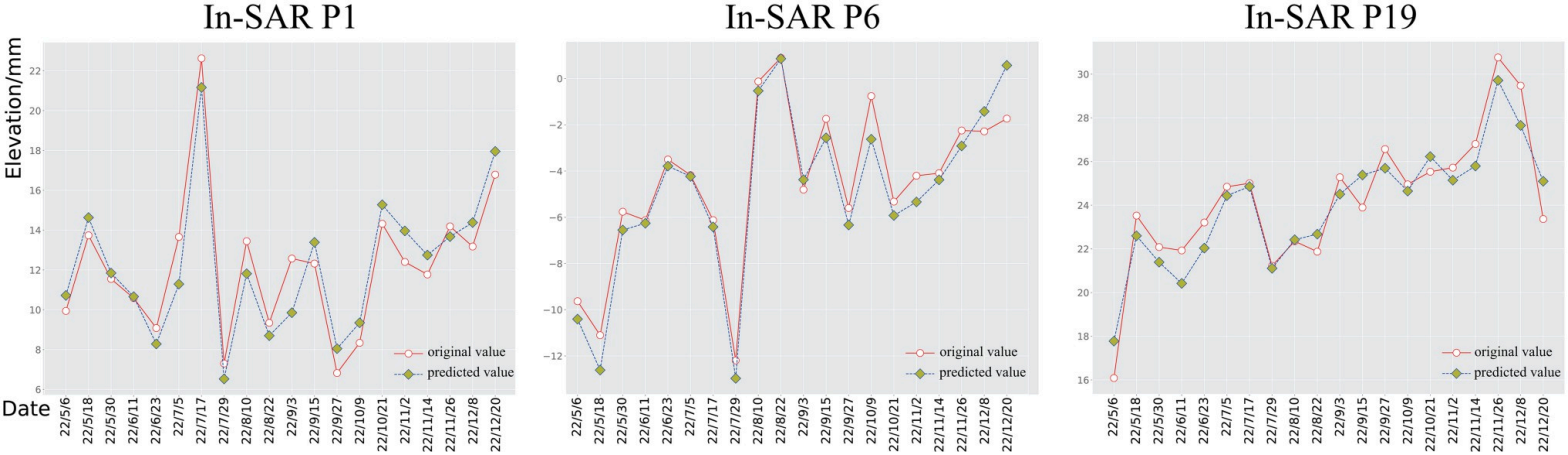
Comparison between predicted and observed values. Time series of subsidence of points *p*_1_, *p*_6_ and *p*_19_ selected from the In-SAR control network.

**Fig 6:**
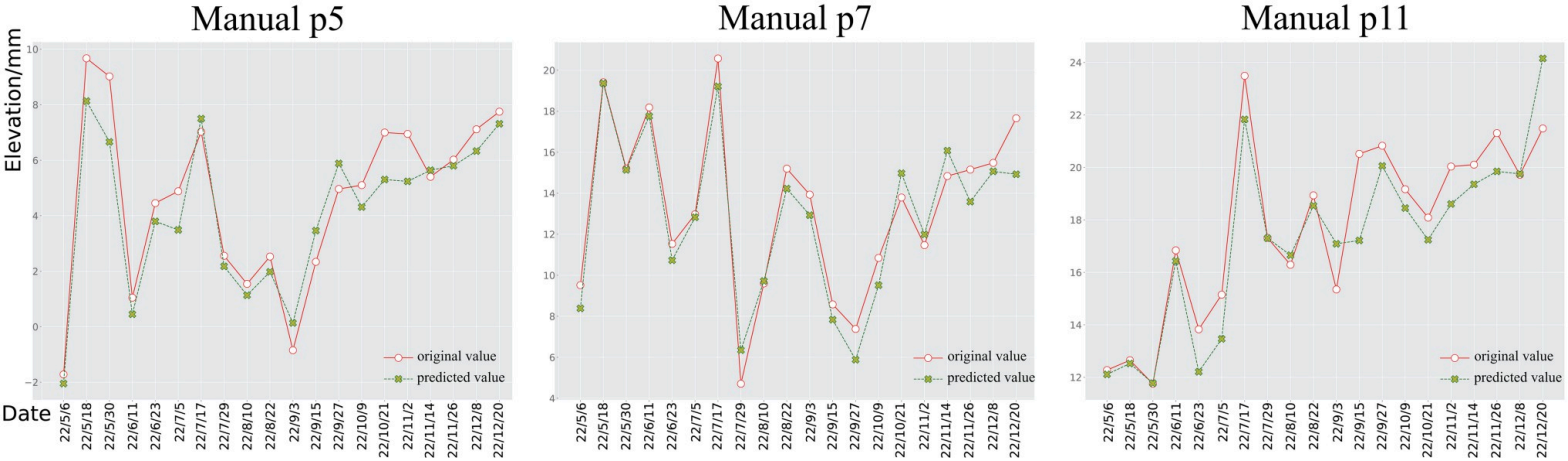
Comparison between predicted and observed values. Time series of subsidence of points *p*_5_, *p*_7_ and *p*_11_ selected from the manual level observation network.

The results in Table 1 show that the machine learning-based approach (LSTM) outperforms traditional methods (ARIMA) in terms of accuracy, MRF-GCN outperforming all other methods in all metrics. Specifically, MRF-GCN outperforms LSTM by 4.83% and 89.19% in the Pearson correlation coefficient (PCC) and coefficient of determination (*R*^2^), respectively. Additionally, MRF-GCN outperforms LSTM by 1.4162*mm*^2^ in terms of mean squared error, indicating a closer fit to the actual data. To visualize how the MRF-GCN prediction results match the true values, we select three points from each of the InSAR-based and manual-based area and plot them as time series diagrams, respectively. As shown in Figs 5 and 6, for the InSAR based control network, points *p*_1_, *p*_6_ and *p*_19_ are selected, and for the manual measurement based control network, points *p*_5_, *p*_7_ and *p*_11_ are selected. It should be noted that these points were chosen randomly just to show the prediction effect of the model, and the time series distribution plots of the remaining points can be fully reproduced by the code in this paper. With these line plots, it can be seen that the prediction results of MRF-GCN fit the real subsidence pattern well. Such prediction results can provide credible support for developing subsidence prevention measures.

**Fig 7:**
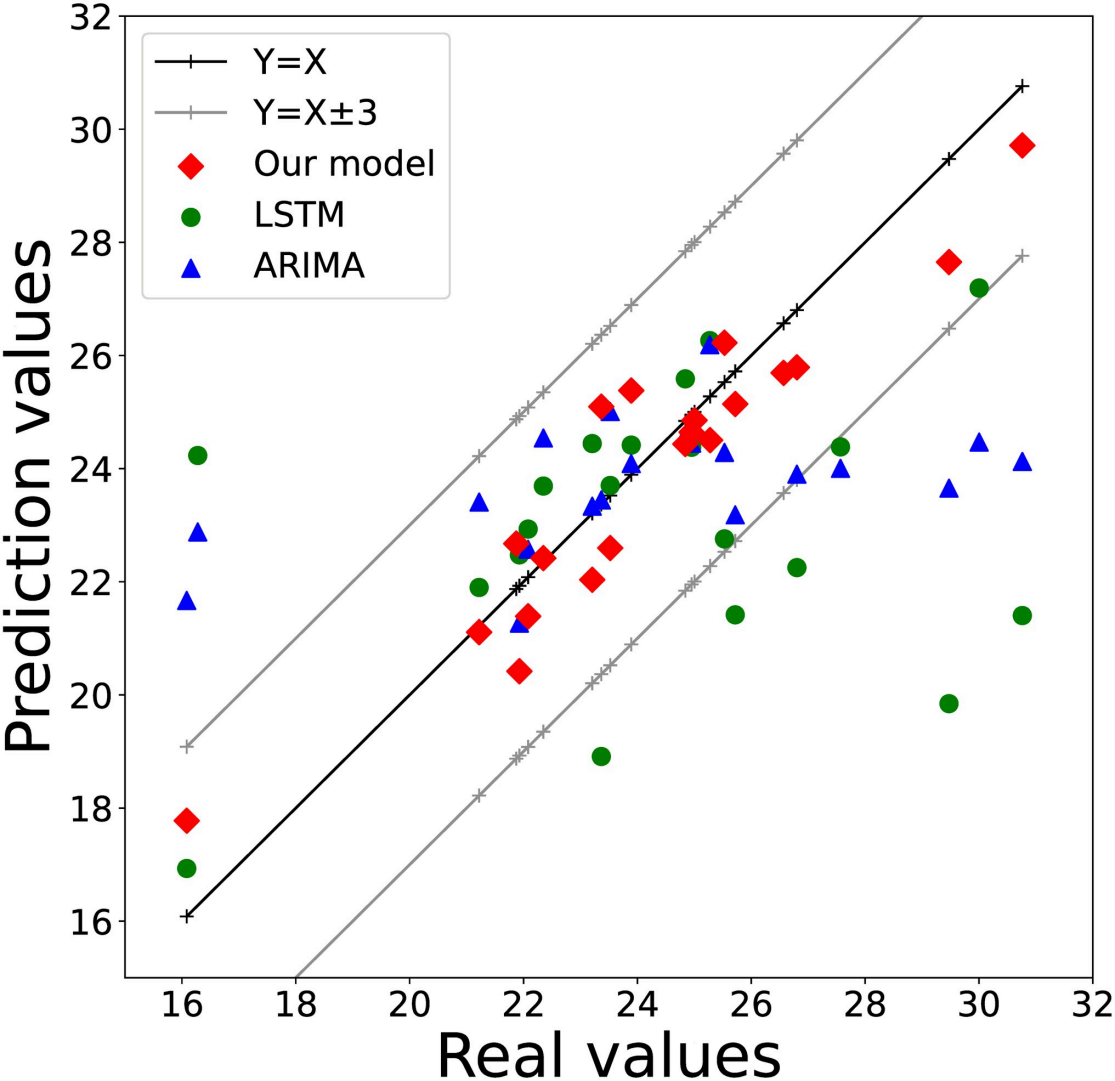
Error distribution. The red, green, and blue scatter points correspond to the error distribution of MRF-GCN, LSTM, and ARIMA, respectively.

To thoroughly investigate the accuracy of the model prediction results, we conducted a visual analysis of the error distributions of MRF-GCN and the other benchmark models. To clearly reflect the prediction effect of different models, we randomly selected a point in Fig 2 under InSAR-based imagery and used different models to predict the settlement change at this point for 20 consecutive time steps and plotted it as a error distribution scatter plot in Fig 7. Specifically, we present the X-Y error distribution, where the actual value of the test set is plotted along the horizontal axis, and the prediction results obtained from MRF-GCN, LSTM, and ARIMA are plotted along the vertical axis, while labeling *Y* = *X* and *Y* = *X* ± 3 on the graph. The red, green, and blue scatter points correspond to the error distribution of MRF-GCN, LSTM, and ARIMA, respectively. It is evident from Fig 7 that the red scatter points are positioned closer to the *Y* = *X* line and exhibit a distribution inside *Y* = *X* ± 3 compared to the green and blue scatter points. This observation implies that the prediction results based on the MRF-GCN model are significantly closer to the actual value of the data than the traditional time series prediction methods, and the prediction results are relatively more stable. Therefore, it can be concluded that the MRF-GCN model outperforms the traditional prediction methods in terms of accuracy and stability.

**Fig 8:**
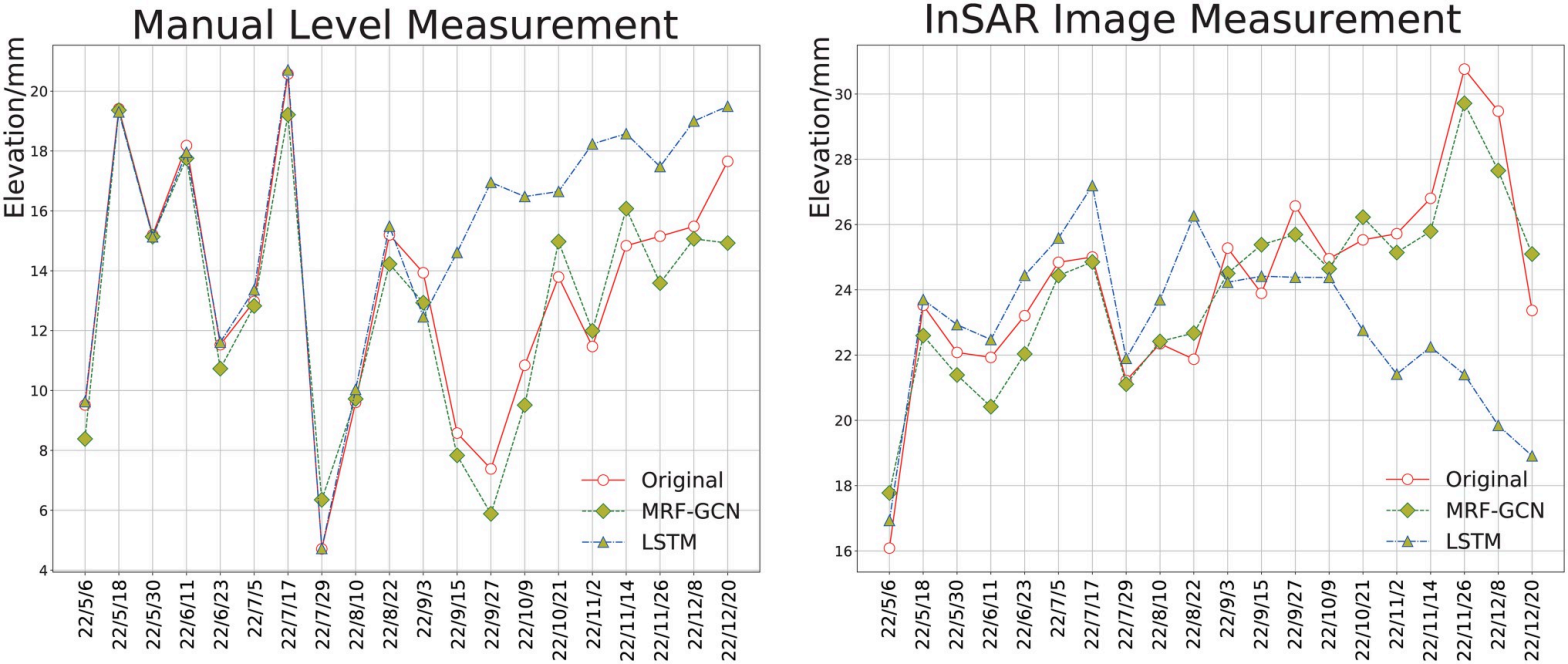
MRF-GCN and LSTM time series comparison.

To provide a comprehensive evaluation of the long-term prediction capability of the proposed model, we conducted a time series comparison between the prediction results of MRF-GCN and LSTM with the actual values of the test set, as illustrated in Fig 8. The analysis of the figure indicates that in the first ten prediction steps, both MRF-GCN and LSTM models exhibit a good fit with the actual data. However, starting from the 11th step, the performance of the LSTM-based model starts to degrade, while the proposed model can still capture the actual data change. This phenomenon can be attributed to the fact that the LSTM model is trained based on a single monitoring point, which only learns the temporal variation pattern of that specific point and lacks the consideration of spatial heterogeneity between different monitoring points. Therefore, the LSTM model fails to incorporate the degree of influence of changes in other monitoring points on the settlement of the current monitoring point, resulting in the model’s early deviation from the actual situation in long-term subsidence prediction. In contrast, our proposed model employs the LSTM module to encode the subsidence sequence and then utilizes the GCN module to learn the interaction law between monitoring points, which effectively enhances the model’s resistance to noise in long-term prediction and achieves better performance. Additionally, Fig 8 also provides a visual illustration of the superiority of our proposed MRF-GCN model in capturing the complex and nonlinear relationship between monitoring points, which is critical for the accurate prediction of long-term subsidence behavior.

**Fig 9:**
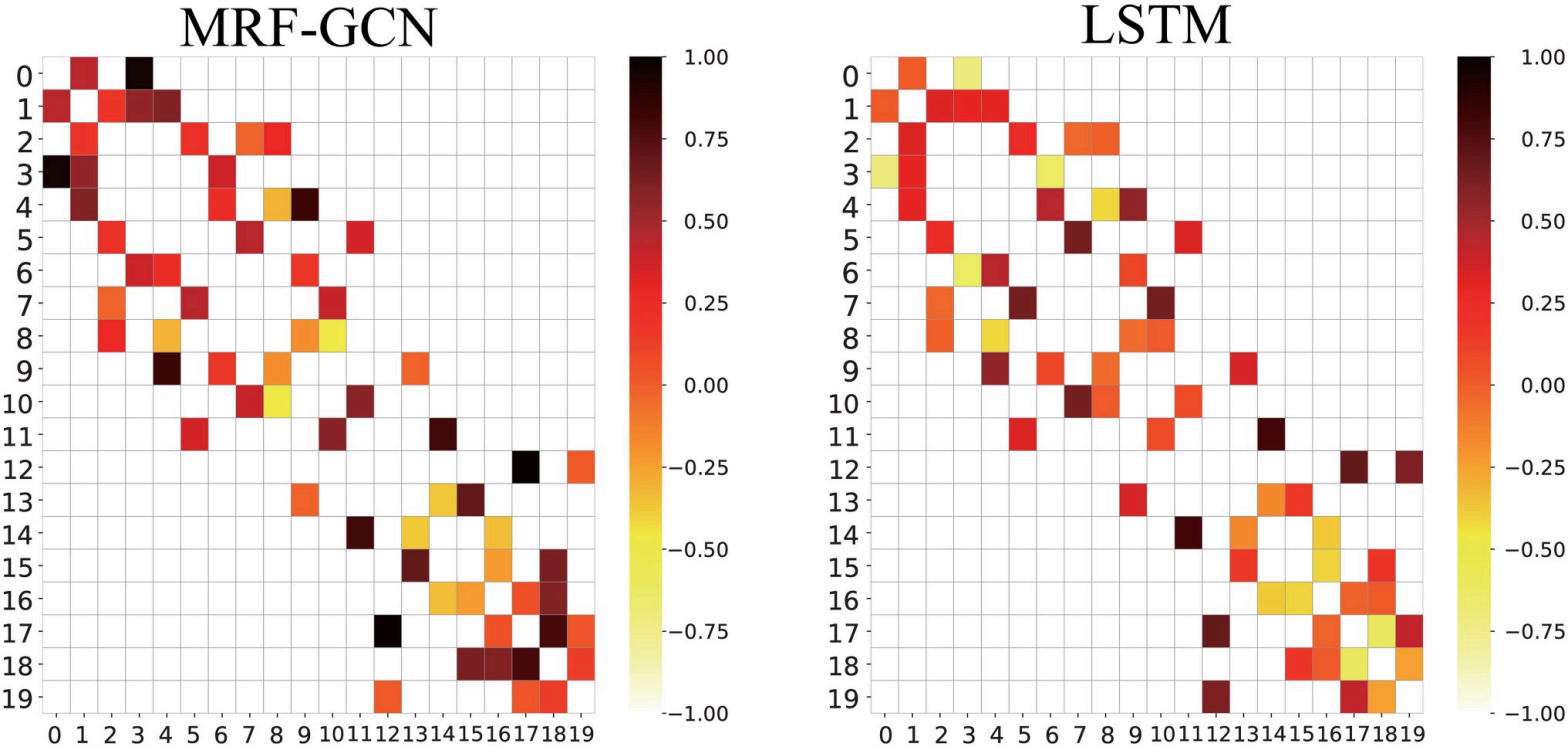
Heatmap of Pearson correlation coefficient. The Pearson correlation coefficient between any two points is indicated by different color shades.

The application of graphical convolutional networks in modeling the subsidence patterns of multiple points can unveil the interplay among the points. The Pearson correlation coefficient (PCC), which ranges from -1 to 1, reflects the degree of correlation between the two vectors. A higher positive absolute value of PCC indicates a more robust correlation and the same trend direction, and vice versa. Considering the predicted data of each point as a vector, the correlation coefficient between two points can reflect the correlation between their subsidence patterns. To examine whether the model can capture the interdependencies among the points after adding the graph convolution, we use the raw data based on InSAR images to generate predictions by the MRF-GCN and LSTM models, respectively compute the PCC between any two points using the prediction sequence. We present the correlation degrees between different points as a 20 × 20 heat map in Fig 9, where the shade of color reflects the strength of correlation. Notably, the point relationships in Fig 9 follow those in Fig 2 based on the control network of InSAR images while ignoring the unconnected points.

Inspection of Fig 9 reveals that the correlation results predicted by MRF-GCN outperform those predicted by LSTM. For example, in the control network, point *p*_4_ is connected to both points *p*_8_ and *p*_9_. In contrast, *p*_9_ is closer to *p*_4_ and is located in a stable geological formation, while *p*_8_ is farther away and located near water. Therefore, *p*_9_ is closer to *p*_4_ in terms of subsidence pattern than *p*_8_ under the same circumstances. In the heatmap, the MRF-GCN based predictions reflect this actual situation well, with the (4,9) grid being significantly darker than the (4,8) grid, while the LSTM-based predictions is unable to achieve this well. These patterns can be accurately learned by modeling the neighboring relationship map using GCN. In contrast, the conventional LSTM prediction model treats the variation of each point in isolation and hence cannot model the mutual influence law between the two points.

## 4 Conclusion

In this paper, we proposed a MRF-GCN model for mining-induced surface subsidence prediction. Unlike previous work, this paper introduced a graph convolutional neural network focusing on the change trend of associated points, and the model can predict the change of current point by learning the subsidence trend of neighboring points. Specifically, to demonstrate how the model works, this paper took the surface subsidence area at the 82/83 mining area of Yuandian No. 2 Mine in Anhui Province as the study area and combined remote sensing technology and manual measurement to obtain subsidence data. These data were used to model with MRF-GCN and to predict the future subsidence trend. The experimental results indicate that the *R*^2^ value of MRF-GCN model was 0.8650, much larger than that of Long-Short Term Memory (LSTM) and other conventional models, while mean square error (MSE) of MRF-GCN model was 1.5989, much smaller than that of LSTM and other conventional models. In a restricted view, in order to explore the prediction performance of the model, we ignore the accuracy requirement of remote sensing data and assume that the subsidence data obtained by multiple Master-image Coherent Target Small-baseline Interferometric SAR method meet the actual situation, but this is not allowed in practical applications, so when using this model to predict the actual subsidence area, remote sensing data that meet the accuracy requirement should be obtained. In a word, the model proposed in this paper can well meet the needs of large-scale surface subsidence prediction, and has the potential to be applied to predict surface subsidence caused by various factors.

## Supporting information

S1 File (ZIP)
